# “Atypical” Mild Clinical Presentation in Elderly Patients With Ruptured Intracranial Aneurysm: Causes and Clinical Characteristics

**DOI:** 10.3389/fsurg.2022.927351

**Published:** 2022-07-08

**Authors:** Dingke Wen, Ruiqi Chen, Tianjie Zhang, Hao Li, Jun Zheng, Wei Fu, Chao You, Lu Ma

**Affiliations:** Department of Neurosurgery, West China hospital, Sichuan University, Chengdu, China

**Keywords:** intracranial aneurysm, elderly patients, aneurysmal subarachnoid hemorrhage, risk factor, prognosis

## Abstract

**Objective:**

Thunderclap-like severe headache or consciousness disturbance is the common “typical” clinical presentation after aneurysmal subarachnoid hemorrhage (aSAH); however, a slowly developing “atypical” clinical pattern, with mild headache, vomiting, or dizziness, is frequently noted in elderly patients. The aim of this study was to evaluate the clinical characteristics of this “atypical” subgroup, as well as related factors associated with the presence of these mild symptoms.

**Methods:**

The data of 176 elderly patients (≥70 years old) with ruptured intracranial aneurysms (IAs) treated at our center from January 2016 to January 2020 were retrospectively collected and analyzed. The patients were divided into “typical” and “atypical” groups based on their initial and development of clinical symptoms after the diagnosis of aSAH. Intergroup differences were analyzed, and factors related to the presence of these two clinical patterns were explored through multiple logistic regression analyses.

**Results:**

Despite significant admission delay (*P* < 0.001) caused by mild initial symptoms with slow development, patients in the “atypical” group achieved better clinical prognosis, as indicated by a significantly higher favourable outcome ratio and lower death rate upon discharge and at different time points during the 1-year follow-up, than the “typical” group (*P* < 0.05). Multiple logistic regression analysis revealed that modified Fisher grade III-IV (OR = 11.182, *P* = 0.003), brain atrophy (OR = 10.010, *P* = 0.001), a larger lesion diameter (OR = 1.287, *P* < 0.001) and current smoking (OR = 5.728, *P* < 0.001) were independently associated with the presence of “typical” symptoms. Aneurysms with wide necks (OR = 0.013, *P* < 0.001) were independently associated with the presence of “atypical” symptoms.

**Conclusions:**

“Atypical” presentations, with mild clinical symptoms and slow development, were commonly recorded in elderly patients after the onset of aSAH. Despite the prolonged admission delay, these “atypical” patients achieved better clinical outcomes than those with “typical” symptoms. Modified Fisher grade (III-IV), current smoking, brain atrophy and larger lesion diameter were factors predictive of “typical” symptoms, while aneurysms with wide necks were independently associated with “atypical” symptoms.

## Introduction

Aneurysmal subarachnoid hemorrhage (aSAH) is a fatal cerebrovascular disease that represents a neurological emergency with potentially devastating consequences (1). aSAH is commonly diagnosed in the working-age population, with half of the patients being younger than 55 years old (2). In recent decades, with the prolongation of life expectancy and improvement of imaging diagnosis, an increasing number of intracranial aneurysms (IAs) have been diagnosed in elderly patients (3). In previous studies, significant differences have been observed in the clinical characteristics between elderly patients and other age groups with IAs (4). In terms of clinical presentation, we observed that nearly half of our elderly patients over the age of 70 years old with ruptured IAs did not present with a common “typical” symptom such as sudden onset of severe thunderclap-like headache or consciousness disturbance (5). In contrast, their clinical presentations were generally “milder” in clinical settings and were characterized by the slow development of headaches, vomiting, or dizziness. To the best of our knowledge, few studies have emphasized such “atypical” symptoms in elderly patients, meaning their clinical characteristics, including demographic information, lesion radiological features, incidence of complications and, most importantly, clinical outcomes, are unknown. The aim of this study was to reveal the clinical characteristics of such an “atypical” symptom subgroup by comparing them with a “typical” symptom group. By exploring potential factors related to the presence of these “atypical” symptoms, the results of our study will be beneficial in improving the understanding of ruptured IAs in elderly individuals.

## Materials and Methods

### Study Design

This was a retrospective study conducted at a single tertiary center that was approved by the institutional review board of West China Hospital. The hospital information system was used to collect the related medical data of elderly patients with IAs from January 2016 to January 2020. The inclusion criteria were as follows: (1) IA as the discharge diagnosis, identified by the key words “intracranial aneurysm”; (2) age ≥70 years; and (3) evidence of rupture of subarachnoid hemorrhage (SAH) or intracerebral hemorrhage (ICH) on initial computed tomography (CT). Two experienced neurosurgeons and two neuroradiologists who were blinded to the study protocol were asked to confirm the diagnoses of the selected patients. Those with an incorrect diagnosis or incomplete medical profiles were excluded.

### Data Collection

Patient baseline information, including age, sex, smoking status, alcohol consumption, history of previous illness; clinical presentations (including symptoms and signs), Glasgow Coma Scale (GCS) scores on admission and upon discharge, Hunt-Hess grade, and duration from ictus to admission were all collected from the hospital information system; neuroradiological data, including modified Fisher grade based on the results of initial head CT, vascular anomalies, brain atrophy, and aneurysm information (size, presence of multiple aneurysms, morphology and location) based on the results of computed tomography angiography (CTA) or digital subtraction angiography (DSA), were also collected. For brain atrophy, we alternatively adopted the ICR (Insular cistern width, Right) and ICL (Insular cistern width, Left) index as brain atrophy index. ICR and ICL have been previously proven strong correlated with aging and cerebral atrophy in literature (6). For a ICR ≥ 7.17 mm, or a ICL ≥ 7.33 mm, the patient would be considered with brain atrophy, otherwise the patient would be considered free from cerebral atrophy lesion (Supplementary Figure 1). Two neurosurgical physicians (RQ C and TJ Z) separately read the CT scans, and dispute was settled by the senior author (LM). Vascular anomalies included fetal-type posterior cerebral artery (FTP) and cerebrovascular atherosclerotic stenosis (CAS), which is characterized by hyperplasia of the vascular wall. Irregular aneurysms were defined as the presence of a daughter sac in addition to the main saccular aneurysm. Wide-neck aneurysms were defined as those with a neck >4 mm in diameter or a dome-to-neck ratio <2. Treatment modalities and the occurrence of complications during hospitalization were tracked in the daily medical records. Clinical outcomes upon discharge were measured by the GCS and modified Rankin scale (mRS) during follow-up, with a GCS score ≥13 and mRS score 0–2 considered to indicate favorable outcomes.

### Grouping

Based on initial presentations and the development of clinical symptoms recorded in the medical record upon the diagnosis of aSAH, the patients were divided into “typical” and “atypical” groups by two neurosurgeons blinded to the study protocol. Patients in the “typical” group were characterized as having sudden onset and rapid development of thunderclap-like headache or unconsciousness disturbance. Those with slow progression of symptoms, such as mild headaches, vomiting, and dizziness, were included in the “atypical” group. Intergroup differences, including patient demographic information, clinical presentations, radiological characteristics, treatment modalities, complications during hospitalization and clinical outcomes, were analyzed according to the study purposes.

### Statistical Analysis

SPSS statistical software (version 22.0; SPSS Inc., Chicago, Illinois, USA) was used for all statistical analyses. The mean ± standard deviation (SD) is reported for quantitative data. Categorical data are expressed as frequencies and percentages. Univariable analyses were conducted using the chi-square test or Fisher's exact tests, Student’s t-tests, and Mann–Whitney U-tests as appropriate. Multiple logistic regression analysis was performed by including potential factors from the univariable analyses to determine independent risk factors related to the presence of “typical” and “atypical” symptoms. Significance was defined as *P* < 0.05, and 95% confidence intervals (CIs) were calculated for each variable.

## Results

### Demographic Characteristics

From January 2016 to January 2020, a total of 2730 patients with IAs were treated in our hospital, including 290 (10.6%) elderly patients (≥70 years old). Among these elderly patients, 176 (60.7%) had ruptured aneurysms and were analyzed in the current study. The mean age of the patients was 74.5 ± 4.8 years, and females accounted for the majority (132 patients, 75.0%). Patient baseline information, including demographic characteristics and history of previous illness, is shown in Table 1. The patients were divided into “typical” (*n* = 90, 51.1%) and “atypical” (*n* = 86, 48.9%) groups based on their initial clinical presentations after the diagnosis of SAH as mentioned above. Compared with the “atypical” group, the “typical” group had significantly higher percentages of patients with daily smoking (14.4% vs. 2.3%, *P* = 0.009) and a history of coronary disease (23.3% vs. 11.6%, *P* = 0.042). The “atypical” group had a significantly higher ratio of patients with anemia (75.6% vs. 60%, *P* = 0.027).

**Table 1:** Baseline information of elder patients with ruptured intracranial aneurysms.

Characteristics	All combined *n* = 176	Typical *n* = 90	Atypical *n* = 86	*P* value*
Age (SD)/years old	74.5 (4.8)	74.5 (4.8)	74.6 (4.9)	0.926
Female	132/176 (75.0)	64/90 (71.1)	68/86 (79.1)	0.223
Smoking, (%)	15/176 (8.5)	13/90 (14.4)	2/86 (2.3)	0.009
Alcohol consumption, (%)	6/176 (3.4)	4/90 (4.4)	2/86 (2.3)	0.720
Hypertension, (%)	144/176 (81.8)	74/90 (82.2)	73/86 (84.9)	0.634
Anemia, (%)	119/176 (67.6)	54/90 (60.0)	65/86 (75.6)	0.027
Diabetes mellitus, (%)	133/176 (75.6)	69/90 (76.7)	64/86 (74.4)	0.729
Hypoproteinemia	130/176 (73.9)	64/90 (71.1)	66/86 (76.7)	0.395
Electrolyte disturbance, (%)	113/176 (64.2)	62/90 (68.9)	51/86 (59.3)	0.185
Hyperlipidemia, (%)	17/176 (9.7)	11/90 (12.2)	6/86 (7.0)	0.239
Pulmonary infection, (%)	80/176 (45.5)	46/90 (51.1)	34/86 (39.5)	0.123
Coronary disease, (%)	31/176 (17.6)	21/90 (23.3)	10/86 (11.6)	0.042
Ischemic stroke, (%)	72/176 (40.9)	33/90 (36.7)	39/86 (45.3)	0.242
Hemorrhagic stroke, (%)	6/176 (3.4)	2/90 (2.2)	4/86 (4.7)	0.637

*Elder patients: age ≥70 years old; Typical, Patients had sudden onset of severe thunderclap-like headache or unconsciousness disturbance after aneurysm rupture; Atypical, Patients with slow progression of symptoms such as mild headaches, vomiting, dizziness after aneurysm rupture; SD, standard deviation.*

* *Differences between Typical group and Atypical group.*

### Clinical Presentations

The patients' clinical manifestations are shown in Table 2. In addition to the initial presence and rapid development of symptoms, the “typical” group had significantly higher percentages of patients with unconsciousness (45.6% vs. 26.7%, *P* = 0.010) and meningeal irritation (58.9% vs. 17.4%, *P* < 0.001) than the “atypical” group. The “atypical” group had significantly higher ratios of patients with dizziness (23.3% vs. 2.2%, *P* < 0.001) and cranial nerve deficits (19.8% vs. 5.6%, *P* = 0.004). In addition, the patients in the “atypical” group had a significantly longer admission delay (493.5 ± 1253.9 vs. 35.0 ± 40.4 h, *P* < 0.001) and higher GCS score (12.5 ± 3.7 vs. 11.3 ± 4.3, *P* < 0.001) on admission. Although the percentage of patents with good clinical presentation (Hunt-Hess grade I-III) showed no significant difference between the two groups (*P* > 0.05), the typical group had a significantly higher percentage of patients with fatal conditions (Hunt-Hess grade V) (8.9% vs. 0%, *P* = 0.014).

**Table 2:** Clinical presentations of elder patients with ruptured intracranial aneurysms.

Characteristics	All combined (*n* = 176)	Typical (*n* = 90)	Atypical (*n* = 86)	*P* value*
Headache, (%)	148/176 (84.1)	77/90 (85.6)	71/86	0.587
Unconsciousness, (%)	64 (36.4)	41/90 (45.6)	23/86 (26.7)	0.010
Vomiting, (%)	118 (67.0)	64/90 (71.1)	54/86 (62.8)	0.240
Dizziness, (%)	22 (12.5)	2/90 (2.2)	20/86 (23.3)	<0.001
Hemiparesis, (%)	20 (11.4)	8/90 (8.9)	12/86 (14.0)	0.290
Seizure, (%)	4 (2.4)	2/90 (2.2)	2/86 (2.3)	0.646
Meningeal irritation, (%)	68 (38.6)	53/90 (58.9)	15/86 (17.4)	<0.001
Cranial nerve deficit, (%)	22 (12.5)	5/90 (5.6)	17/86 (19.8)	0.004
Admission delay (SD)/hours	264.2 (914.0)	35.0 (40.4)	504.1 (1253.9)	<0.001
GCS (SD)	11.9 (4.1)	11.3 (4.3)	12.5 (3.7)	0.048
Good clinical presentation, (%)	130/176 (73.9)	64/90 (71.1)	66/86 (76.7)	0.395
Fatal clinical presentation, (%)	8/176 (4.5)	8/90 (8.9)	0/86 (0)	0.014

*Elder patients: age ≥70 years old; Typical: Patients had sudden onset of severe thunderclap-like headache or unconsciousness disturbance after aneurysm rupture; Atypical: Patients with slow progression of symptoms such as mild headaches, vomiting, dizziness after aneurysm rupture; SD: standard deviation; GCS: Glasgow Coma Score; Good clinical presentation: Hunt-Hess grade (I-III); Fatal clinical presentation: Hunt-Hess grade (V).* *Differences between Typical group and Atypical group.

### Radiological Characteristics

The patients' radiological characteristics are shown in Table 3. Compared with the “atypical” group, the “typical” group had significantly higher percentages of patients with a higher modified Fisher grade (III-IV) (74.4% vs. 46.5%, *P* < 0.001) and brain atrophy (22.2% vs. 5.8%, *P* = 0.002) and a significantly larger mean lesion diameter (5.7 ± 3.4 vs. 4.5 ± 3.2, *P* = 0.007). The “atypical” group had a significantly higher ratio of patients with wide neck aneurysms (44.2% vs. 24.4%, *P* = 0.006).

**Table 3:** Radiological characteristics of elder patients with intracranial aneurysms.

Characteristics		All combined (*n* = 176)	Typical (*n* = 90)	Atypical (*n* = 86)	*P* value*
Modified Fisher grade I-II, (%) I-II		69/176 (39.2)	23/90 (33.3)	46/86 (53.5)	<0.001
Modified Fisher grade III-IV, (%)		107/176 (60.8)	67/90 (74.4)	40/86 (46.5)	-
With irregular aneurysms, (%)		88/176 (50.0)	46/90 (51.1)	42/86 (46.8)	0.763
CAS, (%)		121/176 (68.8)	66/90 (73.3)	65/86 (75.6)	0.733
FTP, (%)		33/176 (18.8)	21/90 (23.3)	12/86 (14.0)	0.111
Brain atrophy		25/176 (14.2)	20/90 (22.2)	5/86 (5.8)	0.002
With multiple aneurysms, (%)		51/176 (29.0)	22/90 (24.4)	29/86 (33.7)	0.175
Lesion diameter (SD)/mm		5.1 (3.3)	5.7 (3.4)	4.5 (3.2)	0.007
With wide neck aneurysms		60/176 (34.1)	22/90 (24.4)	38/86 (44.2)	0.006
Location	ICA, (%)	124/176 (70.5)	60/90 (66.7)	64/86 (74.4)	0.260
	MCA, (%)	38/176 (21.6)	22/90 (24.4)	16/86 (18.6)	0.347
	ACA, (%)	18/176 (10.2)	10/90 (11.1)	8/86 (9.3)	0.883
	Acom, (%)	18/176 (10.2)	11/90 (12.2)	7/86 (8.1)	0.519
	PCA, (%)	2/176 (1.1)	0/90 (0)	2/86 (2.3)	0.457
	PICA, (%)	2/176 (1.1)	2/90 (2.2)	0/86 (0)	0.497
	VA, (%)	5/176 (2.8)	0/90 (0)	4/86 (4.7)	0.118
	BA, (%)	1/176 (0.6)	1/90 (1.1)	0/86 (0)	0.327

*Elder patients: age ≥70 years old; SAH: Subarachnoid hemorrhage; ICH: intracerebral hemorrhage; CAS: cerebrovascular atherosclerotic stenosis; FTP: fetal-type posterior cerebral artery; SD: standard deviation; ICA: internal carotid artery; MCA: middle cerebral artery; ACA: anterior cerebral artery; Acom: anterior communicating artery; PCA: posterior cerebral artery; PICA: posterior inferior cerebellar artery; VA: Vertebral artery; BA: basilar artery.*

* *Differences between Typical group and Atypical group.*

### Treatment, Complications and Clinical Outcomes

The patients' treatment modalities, incidence rates of complications during hospitalization, and clinical outcomes are shown in Table 4. In the comparison between the “typical” and “atypical” groups, no significant difference was observed regarding the distribution of treatment modalities. However, the “typical” group had significantly higher ratios of patients with pulmonary infection (56.7% vs. 37.2%, *P* = 0.010), hydrocephalus (32.2% vs. 16.3%, *P* = 0.014) and thrombosis (15.6% vs. 5.8%, *P* = 0.037). The mean length of stay was also significantly longer in the “typical” group than in the “atypical” group (12.6 ± 13.4 vs. 8.4 ± 7.6 days, *P* = 0.012). Regarding clinical outcomes, the “atypical” group had a significantly higher mean GCS score upon discharge (11.4 ± 4.5 vs. 9.4 ± 4.9, *P* = 0.005) and significantly higher ratios of favorable outcomes and lower death rates at different time points (3 months, 6 months and 12 months) during follow-up (*P* < 0.05).

**Table 4:** Treatment, complications and prognosis of elder patients with intracranial aneurysms.

Characteristics		All combined (*n* = 176)	Typical (*n* = 90)	Atypical (*n* = 86)	*P* value*
Craniotomy, (%)		75/176 (42.6)	42/90 (46.7)	33/86 (38.4)	0.266
Endovascular, (%)		33/176 (18.8)	16/90 (17.8)	17/86 (19.8)	0.735
Conservative, (%)		68/176 (38.6)	32/90 (35.6)	36/86 (41.9)	0.391
Pulmonary infection, (%)		83/176 (47.2)	51/90 (56.7)	32/86 (37.2)	0.010
Hydrocephalus, (%)		43/176 (24.4)	29/90 (32.2)	14/86 (16.3)	0.014
Thrombosis, (%)		20/176 (11.4)	14/90 (15.6)	5/86 (5.8)	0.037
Rebleeing, (%)		14/176 (8.0)	10/90 (11.1)	4/86 (4.7)	0.192
Gastrointestinal bleeding, (%)		8/176 (4.5)	7/90 (7.8)	1/86 (1.2)	0.081
Seizure, (%)		2/176 (1.1)	1/90 (1.1)	1/86 (1.2)	0.497
Blood vasospasm, (%)		4/176 (2.3)	4/90 (4.4)	0/86 (0)	0.141
Intracranial infection, (%)		9/176 (5.1)	5/90 (5.6)	4/86 (4.7)	0.944
Mean length of stay (SD)/day		10.5 (11.1)	12.6 (13.4)	8.4 (7.6)	0.012
Discharge	Mean GCS	10.4 (4.8)	9.4 (4.9)	11.4 (4.5)	0.005
	Favorable, (%)	97/176 (55.1)	38/90 (42.2)	59/86 (68.6)	<0.001
	Unfavorable, (%)	49/176 (27.8)	29/90 (32.2)	20/86 (23.3)	0.247
	Death, (%)	30/176 (17.0)	23/90 (25.6)	7/86 (8.1)	0.002
3 month	Favorable, (%)	87/176 (49.4)	34/90 (37.8)	53/86 (61.6)	0.002
	Unfavorable, (%)	53/176 (30.1)	31/90 (34.4)	22/86 (25.6)	0.200
	Death, (%)	36/176 (20.5)	25/90 (27.8)	11/86 (12.8)	0.014
6 month	Favorable, (%)	75/176 (42.6)	29/90 (32.2)	46/86 (53.5)	0.004
	Unfavorable, (%)	56/176 (31.8)	32/90 (35.6)	24/86 (27.9)	0.276
	Death, (%)	45/176 (25.6)	29/90 (32.2)	16/86 (18.6)	0.038
12 month	Favorable, (%)	72/176 (40.9)	28/90 (31.1)	44/86 (51.2)	0.007
	Unfavorable, (%)	46/176 (26.1)	26/90 (28.9)	20/86 (23.3)	0.395
	Death, (%)	58/176 (33.0)	36/90 (40.0)	22/86 (25.6)	0.042

*Elder patients: age ≥70 years old; Typical: Patients had sudden onset of severe thunderclap-like headache or unconsciousness disturbance after aneurysm rupture; Atypical: Patients with slow progression of symptoms such as mild headaches, vomiting, dizziness after aneurysm rupture; SD: standard deviation; GCS: Glasgow Coma Score; Favorable outcomes: GCS score ≥13 at discharge and Modified Rankin Scale of 0–2 during follow up; Unfavorable outcomes: GCS score <13 at discharge and Modified Rankin Scale of 3–6 during follow up.*

* *Differences between Typical group and Atypical group.*

### Multiple Logistic Regression Analysis

Factors with significant differences in the univariable analyses were considered potential predictive factors for the presence of “typical” or “atypical” symptoms and were included in the multiple logistic regression analysis. As shown in Table 5, after adjustment for confounding factors, modified Fisher grade III-IV (OR = 11.182, 95% CI: 2.219–56.357, *P* = 0.003), brain atrophy (OR = 10.010, 95% CI: 2.629–38.106, *P* = 0.001), a larger lesion diameter (OR = 1.287, 95% CI: 1.134–1.461, *P* < 0.001) and current smoking (OR = 5.728, 95% CI: 2.457–13.353, *P* < 0.001) were independently associated with the presence of “typical” symptoms. Aneurysms with wide necks (OR = 0.013, 95% CI: 0.001–0.142, *P* < 0.001) were independently associated with the presence of “atypical” symptoms.

**Table 5:** Multiple logistic regression analysis for the factors related to the presence of “typical” and “atypical” symptoms in elderly patients with ruptured IAs.

Character	Risk ratio	Lower 95% CI	Upper 95% CI	*P* value
Modified fisher grade III-IV	11.182	2.219	56.357	0.003
Aneruysms with wide necks	0.013	0.001	0.142	<0.001
Anemia	1.973	0.897	4.339	0.091
Coronary disease	1.187	0.370	3.813	0.773
Brain atrophy	10.010	2.629	38.106	0.001
Larger lesion diameter	1.287	1.134	1.461	<0.001
Current smoking	5.728	2.457	13.353	<0.001

*Elder patients: age ≥70 years old; Typical: Patients had sudden onset of severe thunderclap-like headache or unconsciousness disturbance after aneurysm rupture; Atypical: Patients with slow progression of symptoms such as mild headaches, vomiting, dizziness after aneurysm rupture.*

## Discussion

The typical clinical picture of SAH due to an aneurysmal rupture is a sudden, severe headache that reaches, in most cases, its maximum intensity within a few seconds and is often reported by patients as “the worst headache in my life”. It is commonly referred to as a typical “thunderclap” or a “sentinel” headache and may last for a few hours or days (5). Based on our study of IAs in patients over 70 years old, in contrast to the “typical” clinical pattern after an IA rupture, nearly half of the patients presented with “atypical” symptoms, with the slow progression of headache, vomiting or dizziness, leading to a significantly longer admission delay; however, these patients seemed to have better clinical outcomes. To the best of our knowledge, the present study is the first to compare the clinical characteristics of ruptured IA with “typical” and “atypical” symptoms in elderly patients. By revealing the factors associated with the occurrence of these two clinical patterns, the results of this study will aid in obtaining a better understanding of ruptured IAs in elderly individuals.

**Figure 1:**
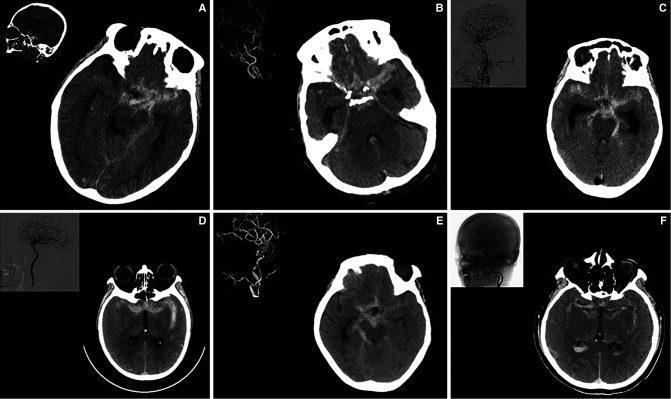
A high modified Fisher grade predicts the development of “typical”symptoms” in elderly patients with ruptured IA. Part **A–F** illustrated 6 individual cases of elderly patients showing increased subarachnoid hemorrhage and expanded subarachnoid spaces. Digital subtraction angiography of related patients was shown on the left upper corner.

We first noted that the elder patients can present as “typical” or “atypical” symptoms in neurological emergencies. It is well known that the meningeal irritation by blood lysis products mainly contributed the pathophysiological mechanism of a “typical” thunderclap-like headache after aSAH (7). Besides, an association with development of vasospasm and worsening of headache was also noted (8). Therefore, a high level Fisher grade, meaning more blood diffused in subarachnoid space, were the major predictors and positively correlated to the presence of “typical” symptoms (Figure 1).

Whereas for elderly individuals over 70, the neuronal sensitivity decreases significantly due to neurodegeneration and demyelination, presented as a decreased neuronal activities after pathological stimuli and an adversely increased tolerance to irritation (9, 10). This implies the elder patients might present with milder clinical symptoms upon similar amount of hemorrhage in subarachnoid space, which validates the high incidence of “atypical” clinical symptoms observed in our present study. This hypothesis was supported by a previous observational study showing high prevalence of unmatched low-grade Hunt-Hess (74%∼89%) score accompanying with high grade Fisher score (79%∼88%) in elderly SAH population (11). Moreover, in comparison to their middle-aged counterparts, the similar low grade “atypical” group of SAH patients only accounted from 12.7% (in younger age group) to a maximum of 40% (not excluding the elder population) in previous cohorts (12–15). Therefore, we speculate the typical clinical symptoms might only occur in elder patients upon a more massive blood diffused in subarachnoid space, in comparison to their younger counterparts (16). The “typical” symptoms in elder patients might in fact much more severe than their younger counterparts.

Furthermore, we find smoking was independently associated with severe “typical” symptoms in elder patients. Smoking has widely been reported as a risk factor related to IA rupture in dose- and duration-dependent manners (17). The main tobacco components and degradation products like nicotine, carbon monoxide, and oxidant compounds can lead to chronic injury of blood vessels (18), like destruction of collagen/elastic fibers, mural cell apoptosis, as well as increased immune cell infiltration (19, 20). Aneurysm enlargement and increased rupture probability have also been noted to be related to current smoking (21). It was hence well-acknowledged that smoking leads to more devastating prognosis after SAH owning to the systemic damage to cerebrovascular condition. In our present study, we also found the “atypical” symptoms, seemingly a protective factor for the elder patients, are not associated with smoking history. This demonstrates an exemption from tobacco damage might contribute to the “atypical” symptoms with less subarachnoid space damage or stimulation. The “typical” severe initial symptoms and worsened outcomes might be partially contributed by a more massive subarachnoid hemorrhage secondary to intensive tobacco-related vessel damage.

Next, we found brain atrophy is commonly diagnosed in elderly patients and pathologically presents as reduced brain volume and an enlarged subarachnoid space (22, 23). Previous study has reported that cerebral atrophy is an independent predictor of unfavorable outcome after intracerebral hemorrhage (24). Currently, the best measurement for brain atrophy would be the reduced brain volume based on 3D reconstructive MRI (6). However, most aneurysmal subarachnoid hemorrhage patients, especially elder patients, were emergently treated without the indication for further MRI scan. CT scan ICR and ICL index were chosen as brain atrophy index.

Based on the results of our study, a correlation between brain atrophy and the intensity of clinical presentation after aneurysm rupture was noted in elderly patients. For patients with brain atrophy, we believe due to decreased adhesion connecting lesions to surrounding tissues and the surrounding lower intracranial pressure (ICP) (25), aneurysms might be more prone to rupture with a sudden fluctuation of blood pressure. In addition, the enlarged subarachnoid space and lower ICP outside the lesion make formation of a localized hematoma difficult, which could be an important factor related to the hemostatic effect in the acute phase after SAH (26). Moreover, due to brain atrophy, bleeding is more likely to spread diffusely in the subarachnoid space on the surface of brain tissues, which could be a major contributor to the high Fisher grade after aneurysm rupture in this age group (27). As mentioned above, the increased bleeding volume and more diffuse blood distribution in the subarachnoid space lead to more severe meningeal irritation and therefore a more severe clinical presentation. This implies a potential correlation between the absence of brain atrophy and “atypical” symptom presence, consequently a better prognosis.

In our present analysis, some radiological features of aneurysms were highly indicative of the symptoms progression in the elderly patients. Among all, the larger aneurysm size (lesion size) was an independent risk factor for the presence of “typical” symptoms with more severe clinical presentation, while the wide-neck presence was confirmed associated with milder “atypical” symptoms. We reckon it was due to the more massive hemorrhage after large aneurysm laceration and milder blood diffusion secondary to wide-neck aneurysm rupture.

It has been reported that aneurysm size is one of the strongest predictors of the risk of rupture (28). In addition, a greater aneurysm size and older age are likely to induce a greater volume of bleeding after subarachnoid hemorrhage with a higher Fisher grade (29). Despite some previous studies based on a middle-aged populations reported that there was no relation between aneurysm size and the volume of subarachnoid blood (30). However, on the basis of significant brain atrophy and vascular degeneration in our elderly cohort, we consider that a larger bleeding volume and more diffuse distribution of SAH could be noted after the rupture of an aneurysm with larger size. Aneurysms with a wide neck, however, are characterized by slower blood flow, less turbulence formation and lower shear stress, which has been widely reported as a factor related to a lower risk of rupture, compare to those with a narrow neck (31). With more stable pressure and hemodynamics across the lesion, rupture of a wide-necked aneurysm might be less likely to cause widespread bleeding in the subarachnoid space (32). This might address its clinical association between milder “atypical” symptoms in some patients.

Previous studies have reported that delayed admission is strongly associated with poor prognosis in SAH patients (33). However, although prolonged admission delay was recorded in the “atypical” group due to mild initial clinical presentation and slow development, their clinical outcomes were significantly better than those of the patients in the “typical” group. For patients in the “typical” group, a higher modified Fisher grade with a lower GCS score on admission and a higher risk of complications, such as pulmonary infections and thrombosis, likely all contributed to the poorer clinical outcomes. These findings suggest that elderly IA patients who have a habit of current smoking and have brain atrophy and a larger aneurysm size are more prone to rupture, with a greater blood volume and more diffuse distribution (higher modified Fisher grade) and are therefore more likely to have a “typical” clinical presentation, with progressive symptoms and worse clinical outcomes. In contrast, patients with wide-necked aneurysms have a higher probability of “atypical” symptoms, with mild symptoms and slow development after rupture; although there may be a delay in hospital admission, the overall prognosis is better.

Regardless of the main findings, our study has some limitations. First, selection bias exists due to the nonrandomized, retrospective study design, which makes it difficult to generalize our results to patients in other areas with different conditions. Second, although there was no significant difference regarding treatment modalities between the two groups, we found that patients in the “typical” group were more likely to receive emergency microsurgical or endovascular surgery due to the rapid development of symptoms. The differences in the timing of surgery as well as preoperative preparation may also be important factors affecting clinical outcomes. However, we did not include such content due to the scope of the database and retrospective design. Therefore, additional well-designed studies that assess additional potential risk factors are required to obtain more robust evidence and verify the results presented in this study.

## Conclusion

In contrast with the “typical” clinical presentation of thunderclap-like severe headache or consciousness disturbance after IA rupture, “atypical” clinical presentations, with mild symptoms and slow development, were commonly recorded in patients in the elderly age group. Despite the prolonged admission delay, elderly patients with “atypical” presentations after aSAH had better clinical outcomes than their “typical” counterparts. Higher modified Fisher grade (III-IV), current smoking, brain atrophy and larger lesion diameter were factors predictive of the presence of “typical” symptoms, while aneurysms with wide necks were independently associated with the presence of “atypical” symptoms.

## Data Availability

The original contributions presented in the study are included in the article/**Suplementary Material**, further inquiries can be directed to the corresponding author/s.
