# Supplementary figures and images for: “Atypical” Mild Clinical Presentation in Elderly Patients With Ruptured Intracranial Aneurysm: Causes and Clinical Characteristics
Source: Front Surg. 2022 Jul 8;9:927351. doi: 10.3389/fsurg.2022.927351 (PMC9304704; doi:10.3389/fsurg.2022.927351)

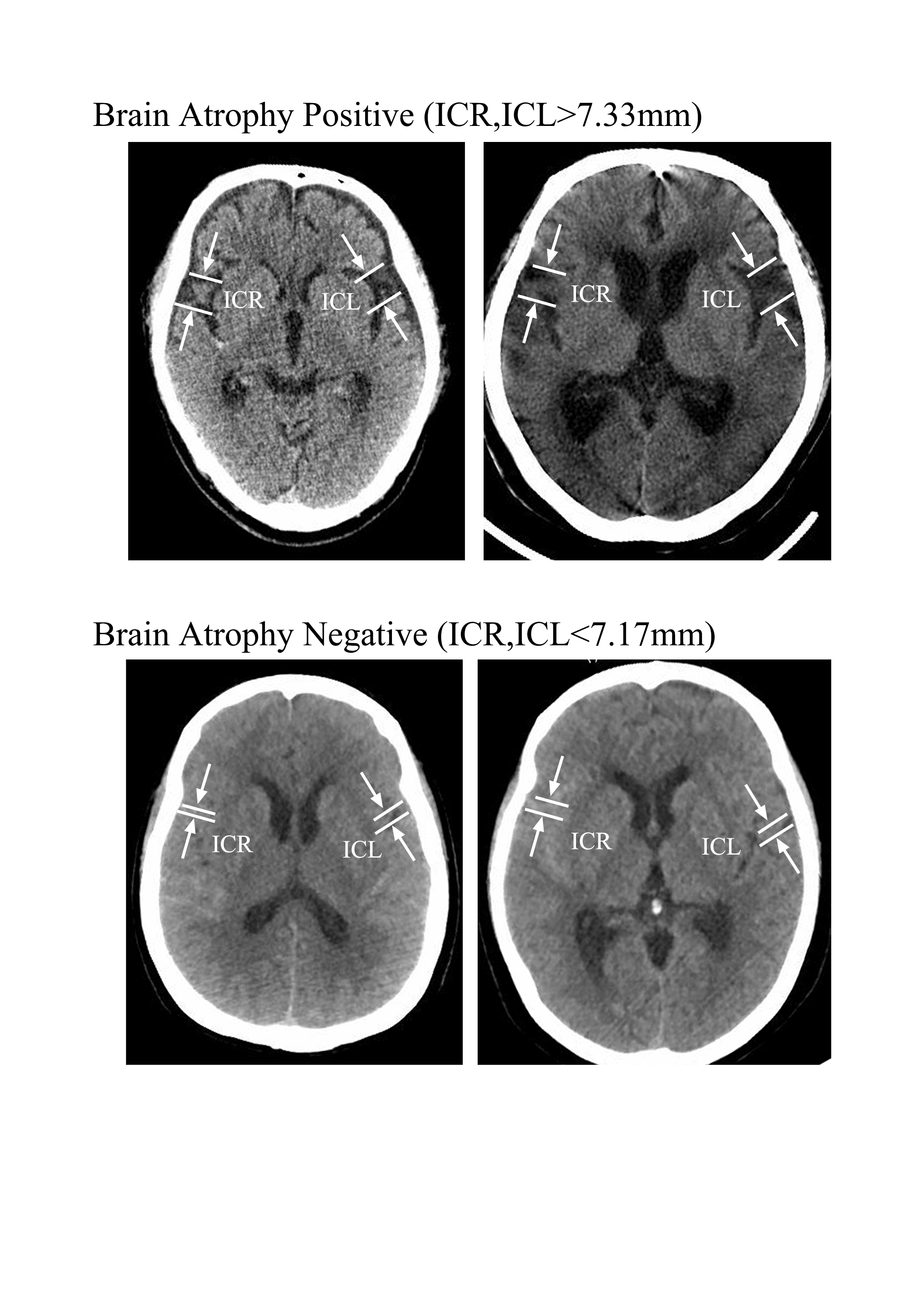

Supplement: Supplementary file 1 [file Image_1_v1.jpeg]
